# Job Demands and Resources and Employee Well-Being in the Chinese Nonprofit Sector

**DOI:** 10.3389/fpsyg.2021.780718

**Published:** 2021-12-20

**Authors:** Guosheng Deng, Chienchung Huang, Shannon P. Cheung, Shaoming Zhu

**Affiliations:** ^1^School of Public Policy and Management, Tsinghua University, Beijing, China; ^2^School of Social Work, Rutgers, The State University of New Jersey, New Brunswick, NJ, United States; ^3^School of Law, University College Cork, Cork, Ireland

**Keywords:** job demands, resources, employee, well-being, foundation, nonprofits, China

## Abstract

Although the nonprofit sector in China has grown substantially in past decades, its future is threatened by high turnover and burnout. It is thus necessary to investigate the factors that contribute to employee well-being (EWB) among nonprofit employees in China. This study used 233 foundation employees in China to examine the effects of job demands and resources (JD-R) on EWB. Estimates produced by regression analyses indicated that job resources (JR) have a strong effect on EWB (Beta = 0.53), as well as on the three EWB subscales (workplace, psychological, and life well-being). While job demands (JD) had no effects on overall well-being, they were negatively associated with workplace well-being (WWB) (Beta = −0.12). Robustness tests were conducted to further examine how JD and JR dimensions affect EWB and its subscales. Based on the findings, we underscore the importance of JR for EWB among foundation employees in China as well as that of implementing interventions that may alleviate the cost of emotional workload as a JD.

## Introduction

With its rapidly growing transitional economy, China has seen a plethora of economic and social changes within the past few decades (Gao, 2017; Garnaut et al., 2018). The transformation from a traditional totalitarian command economy to an open market economy has come with a series of administrative reforms and the expansion of the nonprofit sector (Lan and Galaskiewicz, 2012; Huang et al., 2014; Dong and Lu, 2020; Lu et al., 2020). There are three types of organizations in the Chinese nonprofit sector: foundations, social service organizations (i.e., nonprofit agencies), and social associations (i.e., membership associations; Huang et al., 2014; Liu, 2021). In the past two decades, foundations have increased by seven-fold times, representing the greatest growth among the three types of organizations (China Foundation Development, 2011; Cheng et al., 2020). In 2004, the Chinese government issued its Regulations on Foundation Management, which allowed for the establishment of private charitable foundations. Between 2005 and 2018, the number of foundations across the country grew from just under 1,000 to 7,200, and the number of individuals employed full-time by these foundations nearly tripled from 10,100 to 31,000. Foundation net assets similarly grew substantially, from 4.2 billion RMB (approximately 650.5 million USD) to 159.2 billion RMB (approximately 24.6 billion USD; China Foundation Development, 2011; Cheng et al., 2020). The considerable growth of foundations, however, comes alongside a sharp increase in work demands for foundation employees, who subsequently experience significant burnout and turnover (Tsinghua University Philanthropy Research Institute, 2018; Wen, 2019). In 2017, 27.6% of the employees in foundations and social service organizations left their jobs (Wen, 2019). Another study conducted by Tsinghua University estimated that 20.2% of employees in the nonprofit sector had intentions to resign in 2018. This proportion was highest for employees in social associations (29.0%), followed by those in social service organizations (25.4%) and foundations (15.9%). The main reasons for resignation intention were low wage, limited career perspectives, and high work demands (Tsinghua University Philanthropy Research Institute, 2018). The constant turnover has brought about continuous recruitment pressure and has jeopardized the sustainability of work – and its quality – performed by this sector (Tsinghua University Philanthropy Research Institute, 2018; Wen, 2019). There is a clear need to examine how nonprofit sector employees are affected by their work conditions, which may, in turn, influence their decision to leave their jobs. Thus, we apply the job demands and resources (JD-R) model to investigate JD-R’s relations to employee well-being (EWB) among Chinese foundation employees, a labor force that has grown substantially. The results of such a study can better inform workplace policies to support employees and reduce high burnout and turnover.

## Literature Review and Theoretical Framework

### Employee Well-Being

Employee well-being is referred to the state of employees’ mental and physical health and well-being, resulting from dynamics within and outside of the workplace (Page and Vella-Brodrick, 2009; Bakker, 2015; Zheng et al., 2015). It is a multidimensional construct that encompasses life well-being (LWB), workplace well-being (WWB), and psychological well-being (PWB; Zheng et al., 2015). LWB encompasses life satisfaction and dispositional affect, WWB comprises work satisfaction and work-related affect, and PWB describes an individual’s psychological functioning and their capacities and needs for flourishing (Ryff and Singer, 1996; Page and Vella-Brodrick, 2009; Zheng et al., 2015). The need for a distinct construct of EWB despite the existence of constructs, such as subjective well-being and PWB, comes from the distinctive philosophical origins of these two highly related constructs (Keyes et al., 2002; Jin, 2007), as well as the increasing importance of understanding the well-being of individuals within the context of their organizations (Danna and Griffin, 1999; Robertson and Cooper, 2010; Zheng et al., 2015).

Employee well-being is a key determinant of human functioning and job performance, and it is critical to the development of organizations around the world (Zheng et al., 2015; Bakker and Demerouti, 2018; Koon and Ho, 2021). For example, in one study, the well-being of a sample of 109 employees in a large customer services company was found to be significantly associated with job satisfaction and job performance, even after controlling for several covariates (Wright et al., 2007). Another study similarly found that there was a positive relation between subjective well-being and job performance in a sample of employees (*n* = 170) at a Spanish information and communication technology company (Salgado et al., 2019). Well-being is also a strong and positive predictor of employee retention (Wright and Bonnett, 1992, 2007; Sears et al., 2013). In fact, in one study, Wright and Bonnett (2007) found that a one-point increase in reported well-being doubled the probability of an employee staying in a particular job position. Finally, overall well-being has been found to be a strong and negative predictor of health outcomes, such as emergency room visits and hospital admissions, as well as productivity outcomes, such as unscheduled absences and short-term disability leaves (Sears et al., 2013). For these reasons, EWB has attracted significant attention among scholars of organizational behavior; similarly, organizational leadership has begun to prioritize ways to promote EWB among employees and staff (Danna and Griffin, 1999; Robertson and Cooper, 2010; Zheng et al., 2015; Miao and Cao, 2019). Empirical studies have shown that EWB and satisfaction are related to work conditions, specifically JD-R, in the private sector (Demerouti et al., 2001; Bakker, 2015; Zheng et al., 2015), as well as in the nonprofit sector (Benz, 2005; Knapp et al., 2017; Bastida et al., 2018; Park et al., 2018).

### The Job Demands and Resources Conceptual Model

Demerouti et al. (2001) proposed the JD-R model to explain the processes involved in employee burnout and well-being. In this model, work conditions can be classified as job demands (JD) or job resources (JR). Both JD and JR are multidimensional concepts. Levels of workload, emotional workload, and changes in tasks are key dimensions of JD (Lequeurre et al., 2013; Bakker and Demerouti, 2018). *Workload* indicates an employee’s perception that they have to work to do in the time available to them. *Emotional workload* refers to the effort required to cope with emotions that may be inherent to the job, including organizationally desired emotions, such as remaining calm and empathic, when working with challenging clients. *Changes in tasks* refer to the difficulties posed to employees by changes in job roles and function. Meanwhile, *relationships with colleagues and supervisor* and *information* are important elements of JR. *Relationship with colleagues* describes the workplace’s team atmosphere, as well as an individual’s perception of the potential to receive social support from their co-workers. *Relationship with supervisor* describes how employees perceive their relationship with someone who holds a position above their own. This dimension also asks respondents about the potential to receive social support from their supervisor. *Information* refers to the degree to which individuals may easily or conveniently access information or feedback regarding their job performance (Demerouti et al., 2001; Lequeurre et al., 2013; Bakker and Demerouti, 2018).

JD and JR differentially affect EWB. For example, JD may lead to decreased EWB and burnout through an “health impairment” or “energy-driven” process (Demerouti et al., 2001; Bakker and Demerouti, 2017). Because JD require employees to engage in long-term physical and/or mental efforts, there are associated physiological and/or psychological costs to them. Demerouti et al. (2001) term this “exhaustion.” The JD-R model considers JD as stressors, which, according to Hockey (1993) control model of demand management, activate the body’s performance-protection strategy, which could result in reduced attention and risky decision making (Demerouti et al., 2001). JD has also been found to be associated with a range of negative health outcomes, such as chronic pain (Roelen et al., 2008). By contrast, JR are job aspects that support employees’ work achievements and reduce the costs of JD to employees (Demerouti et al., 2001). Through a motivation process, JR can support EWB and reduce burnout, in turn, promoting job performance (Demerouti et al., 2001; Bakker et al., 2003; Bakker and Demerouti, 2018).

This model has been applied in studies that focus on a number of different work outcomes (e.g., burnout, stress, health, and work engagement) and different occupational groups (e.g., public, private, and nonprofit; Demerouti et al., 2001; Bakker et al., 2003; Hakanen et al., 2008; Schaufeli et al., 2009; Schaufeli and Taris, 2014; Grover et al., 2017; Knapp et al., 2017; Luo and Lei, 2021). Compared to employees in the private and public sectors, nonprofit sector employees tend to have low extrinsic rewards (e.g., fairness of pay and financial well-being) and high JR (e.g., helpful co-workers and supervisors) and job well-being (Benz, 2005; Ariza-Montes and Lucia-Casademunt, 2016; Lee, 2016; Stater and Stater, 2019; Zhou et al., 2020). Studies found that nonprofit employees were more sensitive to JR than employees in the public and private sectors (Ariza-Montes and Lucia-Casademunt, 2016; Bastida et al., 2018; Park et al., 2018; Stater and Stater, 2019). For example, Stater and Stater (2019) used 2002–2014 data from the General Social Survey (GSS) and found that having helpful co-workers and caring supervisors have greater effects on job satisfaction for nonprofit workers than for private and public sector workers. Helpful supervisors discouraged turnover intent to a greater extent in the nonprofit sector than in the for-profit and public sectors. By contrast, extrinsic rewards, such as fairness of pay, were more important to employees in the private and public sectors than for those in the nonprofit sector (Stater and Stater, 2019).

The model has also been applied in several cross-cultural contexts, including the United States (Demerouti et al., 2001), Finland (Hakanen et al., 2006), France (Lequeurre et al., 2013), and China (Hu et al., 2011). Overall, the findings consistently pointed out that JD is associated with low work performance and poor well-being, indicating the health impairment process theorized in the JD-R model. Meanwhile, JR is associated with greater work performance and well-being, indicating the motivation process theorized in the JD-R model (Demerouti et al., 2001; Bakker et al., 2003). Based on the JD-R model, we test a conceptual model involving JD-R and EWB among foundation employees in China. We hypothesized that (1) JD reduces EWB and (2) JR increases EWB. Next, given that JD and JR are characteristics of the work environment, we hypothesized that (3) JD and JR would have the largest effects on WWB than the LWB and PWB. Finally, based on our literature review, we hypothesized that (4) emotional workload in the JD scale and relationship with supervisor in the JR scale would each have the largest effects on EWB and its subscales.

## Materials and Methods

### Data and Sample

The data for the present study came from an anonymous web-based survey conducted by the Social Innovation and Rural Revitalization Research Center (SIRRRC) at the School of Public Policy and Management of Tsinghua University. SIRRRC teamed up with Guangdong Guoqiang Foundation and China Foundation Development Forum to provide trainings for employees from 270 local foundations in March 2021. Given that local and national foundations make up 95.8 and 4.2% of all foundations, respectively (Tsinghua University Philanthropy Research Institute, 2018), SIRRRC randomly selected 12 national foundations and added them into the final sampling of foundations (*n* = 282). SIRRRC sent out invitations to participate in the online survey to 282 foundations on May 20, 2021. SIRRRC sent reminders to complete the survey 7 and 14 days after the initial invitation. Two hundred thirty-three responses were received by June 20, 2021. An informed consent process was implemented prior to the survey. Respondents were informed that their participation was voluntary and that they could choose to stop completing the survey at any time. Participation was compensated with a random draw of a red envelope that contained money (range: 0–18 RMB, 5 RMB, or 1 USD, on average). The research protocol was approved by the research review committee at one of the co-authors’ university in China.

### Measures

The dependent variable, *EWB*, was assessed by the EWB scale, which was developed by Zheng et al. (2015). The scale is comprised of 18 items across three dimensions: WWB, PWB, and LWB. Each dimension is measured by six items, and responses are rated on a seven-point Likert scale, with 1 representing “strongly disagree” and 7 representing “strongly agree.” Higher scores represent greater EWB. Past literature has found this scale to be reliable and valid, with a Cronbach alpha value above 0.80 (Zheng et al., 2015; Miao and Cao, 2019). In this study, Cronbach’s alpha was 0.95. For the WWB, PWB, and LWB subscales, the alpha values were 0.93, 0.89, and 0.92, respectively.

The independent variables, *JD* and *JR*, were measured using items adapted from Questionnaire sur les Ressources et Contraintes Profesionnelles (QRCP), which was developed by Lequeurre et al. (2013). While JD and JR are each comprised of multiple subscales in QRCP, we focus on three dimensions of each. Our JD measure includes *workload*, *emotional workload*, and *changes in tasks*, while our JR measure includes *relationship with colleagues*, *relationship with supervisor*, and *information*. These dimensions were selected based on a review of the literature and the nature of nonprofit work in China. Each of these dimensions is measured with four items. Each dimension has shown strong reliability, indicated by a Cronbach alpha of above 0.80. Example items include: Do you have too much work to do? (workload); Does your work demand a lot from you emotionally? (emotional load); Do you find it difficult to adapt to changes in your tasks? (changes in tasks); Can you count on your colleagues when you encounter difficulties in your work? (relationship with colleagues); Can you count on your superior when you come across difficulties in your work? (relationship with supervisor); Does your work provide you with direct feedback on how well you are doing your work? (information). The questions were translated into Chinese by two Chinese doctoral students in the United States and verified by an American professor whose native language is Chinese. All items were rated on a seven-point Likert scale ranging from 1 (never) to 7 (always). Higher scores indicate greater JD or JR. In this study, Cronbach’s alpha for the JD subscale was 0.82, and for the JR subscale, it was 0.93. The JD and JR scores were calculated by taking the average of all item responses in each scale.

In our model, we control for several demographic and socioeconomic characteristics. These variables included gender (male = 0, female = 1), age, education (below college, college, and above college), and marital status (never married, married, and other). We also controlled for respondents’ position in the foundation: frontline worker, mid-level manager, and senior manager.

### Analytical Approach

Analyses began with descriptive analysis to examine sample characteristics. We also conducted correlation analysis to examine the relations between JD-R and EWB. Then, to estimate the association between the key independent variables – JD and JR – and the dependent variable, EWB, we conducted regression analysis. The framework underlying this study posits that the extent of EWB is determined by JD-R and the demographic and socioeconomic characteristics of nonprofit employees in China. The specification of the analytic model is represented by the following equation:


$$Y_{i}=\alpha_{i}+\beta_{1}*\chi_{i}+\varepsilon_{i}$$

where *Y*i is EWB of the subject *i*; *α_i_* is the individual constant; *β* is a vector of JD-R, and demographic and socioeconomic characteristics of subject *i*; *χ* is a vector of regression coefficients; and *ε_i_* is the cross-section error component. Ordinary least squares (OLS) regression was used for the analyses. All analyses were conducted using STATA software 16.0.

## Results

Table 1 presents the descriptive statistics of the variables. Our sample reported an average score of EWB of 5.0 (SD = 0.9), indicating relatively high EWB compared to that of private sector employees (mean, 4.8; SD = 0.8, *n* = 290, Zheng et al., 2015, two-sample *t* test, 2.69, *p* < 0.001). Among the three dimensions, respondents in this study reported having highest PWB, followed by WWB, and LWB. JD and JR scores averaged at 4.6 (SD = 0.8) and 5.2 (SD = 0.9), respectively. The relative numbers for JD and JR were 3.5 (SD = 1.3) and 5.0 (SD = 1.3) in a military personnel sample (*n* = 490; Lequeurre et al., 2013). Although the samples are not comparable, the results suggest that nonprofit employees in our sample have high JD and high JR. Among the various aspects of JD, workload was highest, followed by emotional workload and changes in tasks. Under JR, employees reported good relationships with their colleagues. This was followed by relationship with supervisor and information. The sample had an average age of 35, and about two-thirds were female. Over half had a college degree (60%) and were married (59%) at the time of completing the survey. 39% of the respondents were frontline workers, while mid- and senior-level managers comprised 31 and 30% of the sample, respectively.

**Table 1:** Descriptive statistics of key variables.

	Mean (SD)
1. Employee well-being (1–7)	5.0 (0.9)
Workplace well-being (1–7)	5.1 (1.0)
Psychological well-being (1–7)	5.4 (0.9)
Life well-being (1–7)	4.7 (1.1)
2. Jon demand (1–7)	4.6 (0.8)
Workload (1–7)	5.4 (1.0)
Emotional workload (1–7)	4.9 (1.0)
Changes in tasks (1–7)	3.6 (1.0)
3. Job resources (1–7)	5.2 (0.9)
Relationship w/Colleagues (1–7)	5.4 (0.9)
Relationship w/Supervisor (1–7)	5.2 (1.1)
Information (1–7)	4.9 (1.1)
4. Female (%)	66.1
5. Age (20–57)	34.9 (7.7)
6. Education (%)	
Below college	15.0
College	60.1
Above college	24.9
7. Marital status (%)	
Never married	34.3
Married	58.8
Other (divorced and widowed)	6.9
8. Type of employee (%)	
Frontline worker	39.1
Mid-level manager	30.9
Senior manager	30.0

*N* = 233. Numbers in brackets show ranges of the variable.

Table 2 presents the results of Pearson’s correlation analysis. JR had a positive correlation with EWB (*r* = 0.59, *p* < 0.001) and with each of the three subscales of EWB. JD did not have any significant correlation with EWB, but two of the JD subscales were significantly correlated with EWB. Changes in tasks was negatively correlated with EWB (*r* = −0.32, *p* < 0.001). Surprisingly, workload was positively correlated with EWB (*r* = 0.20, *p* < 0.01). Workload was also positively correlated with JR (*r* = 0.26, *p* < 0.001). Regression analysis suggests that the positive correlation between workload and EWB was driven by JR. All subscale items were highly correlated with one another, with the exception of workload and changes in tasks.

**Table 2:** Correlations of job demands and resources (JD-R) and well-being.

	1	2	3	4	5	6	7	8	9	10	11	12
1. Job demands	---											
2. Workload	0.76^***^	---										
3. Emotional workload	0.87^***^	0.60^**^	---									
4. Changes in tasks	0.66^***^	0.12	0.38^***^	---								
5. Job resources	−0.03	0.26^***^	0.05	−0.38^***^	---							
6. Relationship w/Colleagues	0.03	0.25^***^	0.06	−0.25^***^	0.84^***^	---						
7. Relationship w/Supervisor	−0.04	0.25^***^	0.07	−0.41^***^	0.90^***^	0.66^***^	---					
8. Information	−0.07	0.17^***^	−0.01	−0.33^***^	0.87^***^	0.59^***^	0.68^***^	---				
9. Employee well-being	−0.08	0.20^**^	−0.07	−0.32^***^	0.59^***^	0.51^***^	0.54^***^	0.50^***^	---			
10. Workplace well-being	0.02	0.17^**^	−0.08	−0.40^***^	0.63^***^	0.51^***^	0.59^***^	0.53^***^	0.91^***^	---		
11. Psychological well-being	−0.10	0.25^***^	0.02	−0.23^**^	0.51^***^	0.47^***^	0.44^***^	0.43^***^	0.88^***^	0.73^***^	---	
12. Life well-being	−0.10	0.13^*^	−0.12	−0.23^***^	0.44^***^	0.38^***^	0.41^***^	0.38^***^	0.88^***^	0.68^***^	0.64^***^	---

*N* = 233.;

* *p* < 0.05;

** *p* < 0.01 and

*** *p* < 0.001.

In Table 3, we present the standardized estimates of EWB, produced by OLS regression. We tested two models. In the first, only demographic and socioeconomic characteristics of the employees were included. In the second model, we added JD-R. The adjusted r-square of Model 1 was 0.14, while the inclusion of JD-R in Model 2 yielded an adjusted r-square value of 0.41, suggesting the importance of JD-R for EWB. In Model 1, educational attainment and employee type had significant effects on EWB. Compared to employees who had below a college education, employees who had obtained a college degree had 0.46 SDs lower EWB. Similarly, compared to senior managers, frontline workers and mid-level managers had 0.46 SDs lower EWB.

**Table 3:** Regression analysis of employee well-being (EWB).

	Model 1			Model 2		
	Beta	SE	*p*	Beta	SE	*p*
Job demands	---	---		−0.07	0.06	
Job resources	---	---		0.53	0.05	***
Female	0.01	0.11		0.01	0.10	
Age	0.10	0.01		0.10	0.01	
Education – college degree	−0.46	0.16	**	−0.16	0.13	*
Education – above college degree	−0.06	0.18		−0.02	0.15	
Never married	−0.04	0.13		−0.02	0.11	
Frontline worker	−0.47	0.14	**	−0.13	0.12	
Mid-level manager	−0.46	0.14	**	−0.20	0.12	**
Adjusted R-square	0.14			0.41		

*N* = 233.

* *p* < 0.05;

** *p* < 0.01 and

*** *p* < 0.001.

In Model 2, JR had a strong and positive effect on EWB, while JD had no significant effect on EWB. An increase of one SD in JR was associated with a 0.53-SD increase in EWB. The findings do not support hypothesis 1 but confirm hypothesis 2. In this model, the estimates of educational attainment and employee type decreased considerably, suggesting that the effects found in Model 1 were driven by JR.

The effects of JD-R were further examined by regressing the three subscales of EWB onto JD-R. The results of this analysis are presented in Table 4. These analyses mirror those that were done to produce the results of Table 3, however, rather than using EWB as the dependent variable, we used each of the EWB subscales (WWB, PWB, and LWB) as dependent variables. JR had significant and positive effects WWB (Beta = 0.56), PWB (Beta = 0.48), and LWB (Beta = 0.38). JD had no significant effect on PWB nor on LWB, but it did have a significant and negative effect on WWB (Beta = −0.12, *p* < 0.05). These findings support hypothesis 3.

**Table 4:** Regression analysis of EWB subscales.

	Workplace WB			Psychological WB			Life WB		
	Beta	SE	*p*	Beta	SE	*p*	Beta	SE	*p*
Job demands	−0.12	0.06	*	0.03	0.07		−0.09	0.08	
Job resources	0.56	0.06	***	0.48	0.06	***	0.38	0.07	***
Female	−0.04	0.11		0.01	0.10		0.07	0.13	
Age	0.07	0.01		0.09	0.01		0.04	0.01	
Education – college degree	−0.15	0.15	*	−0.10	0.15		−0.17	0.18	*
Education – above college	−0.06	0.16		0.01	0.16		0.00	0.20	
Never married	0.03	0.11		0.08	0.12		0.07	0.14	
Frontline worker	−0.15	0.14	*	−0.03	0.14		−0.15	0.16	
Mid-level manager	−0.18	0.13	**	−0.13	0.13		−0.22	0.16	**
Adjusted R-square	0.44			0.29			0.27		

*N* = 233.

* *p* < 0.05;

** *p* < 0.01 and

*** *p* < 0.001.

Lastly, we examined the effects of individual JD and JR items on overall EWB and each of the subscales. In the regression analyses used to produce the results displayed in Table 5, the independent variables were the individual JD and JR items. Each row of Table 5 represents an individual iteration of regression analysis. The results indicate that the individual JR items had significant and consistent effects on EWB and each of the three subscales. Relationship with supervisor tended to have a slightly higher effect on WWB (Beta = 0.53) than did relationship with colleagues (Beta = 0.45), but the latter had a larger effect on PWB (0.43) than did the former (0.40). Emotional workload had a significant and negative effect on EWB (Beta = −0.11), WWB (Beta = −0.12), and LWB (Beta = −0.15), while changes in tasks had a negative effect on WWB (Beta = −0.18). These findings confirm hypothesis 4.

**Table 5:** Robustness tests of EWB regressed onto JD-R items.

	Employee WB			Workplace WB			Psychological WB			Life WB		
	Beta	SE	*p*	Beta	SE	*p*	Beta	SE	*p*	Beta	SE	*p*
Job demands	−0.07	0.06		−0.12	0.06	*	0.03	0.07		−0.09	0.08	
Workload	0.03	0.04		−0.01	0.05		0.11	0.05		−0.01	0.06	
Emotional workload	−0.11	0.04	*	−0.12	0.05	*	0.00	0.05		−0.15	0.06	***
Changes in tasks	−0.10	0.05		−0.18	0.05	**	−0.04	0.05		−0.05	0.07	
Job resources	0.56	0.06	***	0.56	0.06	***	0.48	0.06	***	0.38	0.07	***
Relationship w/Colleagues	0.44	0.05	***	0.45	0.06	***	0.43	0.06	***	0.31	0.07	***
Relationship w/Supervisor	0.48	0.04	***	0.53	0.05	***	0.40	0.05	***	0.35	0.06	***
Information	0.44	0.05	***	0.47	0.05	***	0.40	0.05	***	0.31	0.06	***

*N* = 233.

* *p* < 0.05;

** *p* < 0.01 and

*** *p* < 0.001.

## Discussion

The descriptive statistics show that foundation employees in our sample had high JD, especially workload and emotional workload. This is consistent with previous findings from other studies (Tsinghua University Philanthropy Research Institute, 2018; Wen, 2019). Despite the substantial expansion of the nonprofit sector in China over the past decade, a majority of the organizations are relatively small, with an average of only three full-time staff (Tsinghua University Philanthropy Research Institute, 2018). Foundation employees are responsible for a full range of activities, as a majority of foundations in China are both operating and grant-making foundations (Huang et al., 2014; Cheng et al., 2020). This means that staff must independently plan projects, implement them, and evaluate them, while also writing grant proposals, managing human resources, and performing administrative tasks. They also need to engage with fund management and plan and execute fundraising activities (Tsinghua University Philanthropy Research Institute, 2018). With limited staff and a range of tasks to perform, it is unsurprising that the employees in our sample reported high workload and emotional workload. On the other hand, the employees also reported high JR, especially in relationships with their colleagues and supervisor, which have both shown strong effects on EWB and work engagement (Bastida et al., 2018; Park et al., 2018; Stater and Stater, 2019).

The results of the OLS regression were in line with the hypothesized dual processes of health impairment and motivation of JD-R among nonprofit workers in China. The magnitude of the estimates produced by our analyses suggests that JR have a greater effect on the EWB of Chinese nonprofit employees than do JD, which is consistent with literature that nonprofit employees were more sensitive to JR than employees in public and private sectors (Stater and Stater, 2019). JR had a positive association with EWB, which indicates that, through the motivation process, nonprofit employees may be able to reduce the physiological and psychological costs of JD, subsequently maintaining or improving their own well-being (Bastida et al., 2018; Stater and Stater, 2019). By contrast, although our overall measure of JD had no effect on EWB, further analyses indicated that JD had negative effects on WWB, particularly JD aspects, such as emotional workload and changes in tasks. Emotional workload similarly had a negative effect on LWB.

Overall, these findings are consistent with and expand upon previous findings with the JD-R model in other occupational groups, showing that JD-R are important predictors of EWB (Hakanen et al., 2008; Zheng et al., 2015; Bakker and Demerouti, 2018; Koon and Ho, 2021). This study provides support for relations between JD and WWB and LWB as well as between JR and EWB in a sample of nonprofit employees in China. This is particularly significant, given the quickly rising rates of nonprofit sector development, burnout, and turnover in China. Thus, this context provides guidance for both future practice and research.

The findings of this study have practice implications. Given that this sample reported, on average, high JD, and given the negative relations between JD and WWB, foundation leadership in China needs to be cognizant of the JD projected onto their employees, particularly in terms of emotional workload and changes in tasks. Employers need to consider the implementation of interventions that have been shown to improve mental health and well-being while reducing emotional workload. For example, a considerable body of literature supports the effectiveness of mindfulness-based stress reduction (MBSR), mindfulness-based cognitive therapy (MBCT), and mindfulness-based interventions (MBI) on EWB (Mellor et al., 2016; Lomas et al., 2018; Slutsky et al., 2018; Guidetti et al., 2019). Although the sample did not report high scores for changes in tasks, changes in tasks do have a negative effect on WWB. As such, employers need take this account by clearly delineating employees’ role responsibilities in advance and without sudden changes. In the current study, the sample reported high JR, which had positive effects on EWB. Foundation leadership should continue to maintain supportive work environments for their employees, emphasizing relationships with supervisor and co-workers, as both were positively associated with EWB. Since information is also important for increasing EWB, employers are encouraged to provide direct and frequent feedback regarding employee work performance.

Due to resource limitations, this study focused on foundation employees, a labor force which has grown significantly in the past two decades. Still, research has shown that employees in other types of nonprofit organizations (i.e., social associations and social service organizations) have greater intention to resign from their positions (Tsinghua University Philanthropy Research Institute, 2018). Thus, it is important for future studies to examine the JD-R and EWB among employees in these organizations. This may allow for better understanding of their work conditions, which can then inform the development of policies and interventions for these employees.

While we have discussed the practice implications of our findings, this study must also be considered within the context of its design limitations. For example, since we used cross-sectional data for our analyses, we may only approximate associative relations among JD, JR, and EWB, rather than causal relations. To better approximate causal relations among these variables, future studies should implement longitudinal experimental design. Next, there are likely a number of unobserved variables that may have contributed to our estimates. While we controlled for demographics and socioeconomic characteristics of our sample, there are still other variables that may affect EWB. Indeed, one study by Craig and Kuykendall (2019) found that supportive non-work friendships can affect individuals’ well-being at work. Future studies might consider whether the relations between JD-R and EWB continue to persist even after controlling for external friendships. Another limitation of this study is that data were collected from subjects, who self-reported JD-R and EWB. Self-reporting can lead to reporting errors – unintended and intended. For example, although the survey completed by respondents was anonymous, participants may have underreported JD while overreporting JR and EWB to better save their employers’ face (Chiu and Kosinski Jr., 1995). Thus, future studies may implement data triangulation by collecting information from colleagues, employers, and family members. Finally, it must be noted that the foundation employees who comprised the study sample were also employees who were in the midst of participating in an online training *via* Tsinghua University in 2020. Thus, the extent to which these findings are generalizable to all nonprofit employees in China is unknown and requires further investigation.

## Conclusion

This study analyzed data collected from 233 nonprofit employees in China to investigate the extent to which JD-R affect EWB. The findings support the existing body of cross-cultural research on JD-R and EWB, indicating that in our sample of Chinese nonprofit employees, emotional workload was negatively associated with EWB, while JR were positively associated with EWB. The results underscore the importance of reducing emotional workload and increasing JR for nonprofit employees in China to improve EWB and to mitigate turnover and burnout.

## Data Availability Statement

The raw data supporting the conclusions of this article will be made available by the authors, without undue reservation.

## Ethics Statement

The studies involving human participants were reviewed and approved by Research Review Committee, School of Public Policy and Management, Tsinghua University. Written informed consent for participation was not required for this study in accordance with the national legislation and the institutional requirements.

## Author Contributions

GD and CH: conceptualization, resources, and investigation and data curation. GD, CH, SC, and SZ: methodology and software, validation, formal analysis, and writing – original draft preparation. All authors contributed to the article and approved the submitted version.

## Conflict of Interest

The authors declare that the research was conducted in the absence of any commercial or financial relationships that could be construed as a potential conflict of interest.

## Publisher’s Note

All claims expressed in this article are solely those of the authors and do not necessarily represent those of their affiliated organizations, or those of the publisher, the editors and the reviewers. Any product that may be evaluated in this article, or claim that may be made by its manufacturer, is not guaranteed or endorsed by the publisher.
